# Engineering Dielectric Materials for High-Performance Organic Light Emitting Transistors (OLETs)

**DOI:** 10.3390/ma14133756

**Published:** 2021-07-05

**Authors:** Caterina Soldano

**Affiliations:** Department of Electronics and Nanoengineering, School of Electrical Engineering, Aalto University, Tietotie 3, 02150 Espoo, Finland; caterina.soldano@aalto.fi

**Keywords:** organic light emitting transistor (OLET), gate dielectrics, low-bias transistors, insulating layer, high-*k* dielectrics, high-*k* oxide, high-*k* polymer, light manipulation

## Abstract

Organic light emitting transistors (OLETs) represent a relatively new technology platform in the field of optoelectronics. An OLET is a device with a two-fold functionality since it behaves as a thin-film transistor and at the same time can generate light under appropriate bias conditions. This Review focuses mainly on one of the building blocks of such device, namely the gate dielectrics, and how it is possible to engineer it to improve device properties and performances. While many findings on gate dielectrics can be easily applied to organic light emitting transistors, we here concentrate on how this layer can be exploited and engineered as an active tool for light manipulation in this novel class of optoelectronic devices.

## 1. Organic Light Emitting Transistor as a New Organic Light Emitting Platform

Organic semiconductor-based devices such as organic light emitting diodes (OLEDs), solar cells, memories and organic field-effect transistors (OFETs) are expected to reduce fabrication costs and enable novel functionalities with respect to devices and structures based on conventional inorganic materials [1,2,3]. In the last few years, organic light emitting transistors (OLETs) have been increasingly gathering interest within the scientific and technological community since they combine in the same device, the function of an electrical switch (transistor with modulation of the channel conduction) with the capability of generating light under appropriate bias conditions [4,5].

**Figure 1:**
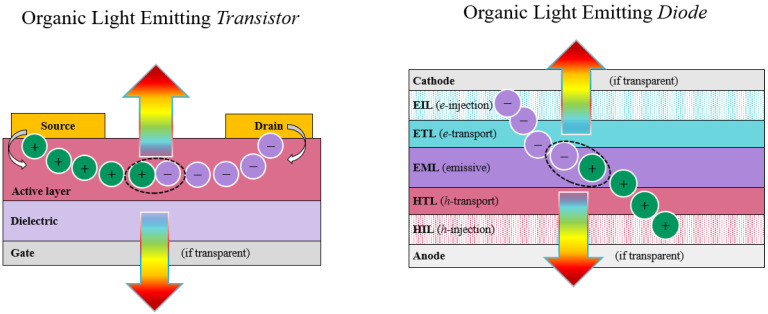
OLET vs. OLED device architectures. Simplified schematic drawings of (**left**) organic light emitting transistor and (**right**) organic light emitting diode structures. OLET is a three-terminals device, separated by a dielectric layer and an active organic material, with the charge transport dominated by lateral field-effect mechanism. OLED is based on a vertical stacked of different organic layers sandwiched between two electrodes, cathode and anode, and transport is dominated by tunnelling through adjacent layers. Each layer within the stack has a specific role (e.g., HIL: hole-injection layer, HTL: hole-transport layer, EML: emissive layer, where light emission occurs, ETL: electron-transport layer, EIL: electron-injection layer).

Organic light emitting transistors are intrinsically very different from more well-known diode counterpart in terms of their structures and operation, mainly in the planar and vertical device geometry (transistor vs. diode, respectively) and corresponding charge transport (lateral *field-effect* vs. vertical bulk), as shown in Figure 1. Besides these fundamental differences, OLET scientific community can nevertheless benefit from extensive research in the OLED field, from which materials and techniques can be “borrowed” to some extent. This device platform has been investigated for about two decades now and has surely profited from extensive knowledge and research work done in the OLED field.

Electroluminescence in these devices can be obtained using either polymers or small molecules. On one hand, conjugated polymers with at least one chain of alternating double- and single bonds, leading to π-bond delocalization, have semiconducting properties as well as they are capable of absorbing sunlight, creating photogenerated charge carriers and transporting these charges [6]. On the other hand, small molecules have become attractive due to their simple and well-defined molecular structure, along with specific semiconducting behaviors (high-mobility organic semiconductors, OSCs) [7]. Small molecules are mainly sublimated in high vacuum, which allows for a higher degree of control over morphology, molecule packing and, thus, on the overall functional films and device properties.

Balance between holes and electrons transport regimes through appropriate gate bias leads to light emitted within the OLET channel; thus, both top and bottom emission (for transparent gate electrode and substrate) can be achieved in these devices. Spatial localization of exciton formation in the transistor favors an effective separation between the exciton population and the charge carriers, thus preventing quenching phenomena.

Organic light emitting transistors can be fabricated in many shapes and geometries and on arbitrary substrates, require less number of layers compared to an OLED, are less sensitive to pinholes and shorts thanks to the presence of the dielectric layer and show higher brightness with both top and bottom emission. Being a voltage-driven device, OLET is potentially less power consuming and easier to be integrated in more complex device architectures; its operating bias is compatible with commercial IC and ultimately it can potentially lead to reduced fabrication cost and increased yield when transferred to industrial and manufacturing processes. OLET can be controlled and driven by any type of driving TFT technology, including “less performing” OTFTs, making them suitable for flexible and wearable electronics applications. In this perspective, OLET indeed represents an alternative and complementary technology platform in the area of (organic) light emitting devices.

Table 1 summarizes the main features of organic light emitting transistors, and for comparison, organic light emitting diodes in terms of charge transport, device architecture and light emission.

OLETs have been demonstrated to have higher external quantum efficiency (EQE) intrinsic of the device structure, with performances outperforming equivalent OLEDs [8], and have higher current densities (1–10 A/cm^2^, for a 1 nm-thick layer) compared to OLEDs (10^−3^–10^−2^ A/cm^2^). Further, the planar device structure along with the two-fold function of light emission and switching renders the OLET an ideal candidate to develop next-generation (flexible) displays [9]. In this field in particular, organic light emitting transistors can introduce some important characteristics:

(i) Transparent displays, which are of fundamental interest in application fields such as augmented reality and automotive, wearable goggles for biomedical use, etc.;
(ii) High degree of integration with various optically active device to manipulate light in more complex architectures;
(iii) Less stringent requirement at the backplane level, in fact being OLET a voltage-driven device, it does not require high-performance driving transistors such as those based on oxides or polycrystalline silicon (LTPS). Organic thin film transistors (OTFTs) fully satisfy requirements to pilot an OLET;
(iv) Simplified pixel architecture, where the inherent capacitance of the organic light emitting transistor can be engineered to accomplish the pixel memory function;
(v) Aperture ratio (defined as the ratio between the area of light emission and the total area of the pixel) of approximately ≈80% [10], readily fulfilling display requirements.
(vi) Potentially pinhole- and shorts-free, given the device intrinsic architecture and the presence of dielectric layer, with the net result of improving yields in production line and reducing manufacturing cost.

Achieving high-performance and highly efficient organic light emitting transistors is the driving force in many studies in the field; integrating high-mobility OSCs and reducing operating voltages to improve brightness levels and power consumptions are key factors towards the full development and implementation of OLET technology platform.

Microelectronics industry (mainly based on Si and other inorganic semiconductors) has seen the successful and extensive use of oxides such as SiO_2_ as gate dielectric in many transistors development and production lines; nevertheless, SiO_2_ has reached its physical limitations [11]. The interest in alternative dielectric materials is mainly two-fold: (i) technological, driven by the continuous demanding reduction of operation voltage essential for consumer electronics applications and (ii) market-driven, where reliable and cheap fabrication processes are highly desirable. A key role in the quest for low-bias applications is played by the high permittivity (high-*k*) dielectrics, with majority of the work devoted to inorganic field-effect transistors [12] and in more recent years to organic FETs [13]. Use of high-*k* dielectrics in organic light emitting transistors is yet largely unexplored, although the overall improvement of its optoelectronic characteristics is likely to be confirmed based on knowledge and studies on (organic) field-effect transistors.

The present Review mainly discusses the role played by the dielectric layer within an organic light emitting transistor platform. First, we will describe the main characteristics and working principle of the OLET; then, we will focus on (i) the role played by high-*k* dielectric layers in achieving high performances devices and (ii) possible new routes to engineer functional dielectric layer to manipulate, and possibly enhance, the light emitted within the device itself. The majority of findings reported for dielectric materials for organic field-effect transistors can be in most cases applied directly to organic light emitting transistors; in this manuscript, we will only refer to organic light emitting transistors, and in all other cases, the Reader will be directed to more specific and relevant available literature.

## 2. Organic Light Emitting Transistors: Main Concept and Mechanism

### 2.1. Building Blocks

Similar to an (organic) field-effect transistors, organic light emitting transistors share the same conventional structure and device structure (as shown in Figure 1), which includes three electrodes and two additional layers. Below, these are briefly summarized:

(a) Three electrodes: source, drain and gate
  - Gate electrode (G) can be either a metal or a transparent conductive oxide. Optical transparency in the visible range allows to extract the light also through the gate, enabling both top and bottom emission. Nowadays, the most used material as transparent conducting electrode is indium-tin-oxide (ITO). However, ITO is currently facing a number of challenges, mainly due to the dramatic price fluctuations as a result of the limited amount of available indium and to its intrinsic rigidity and brittleness upon bending [14]. This has encouraged a broad search for alternative transparent and conductive electrode materials, including metallic nanowires [15], carbon nanotubes [16,17], conductive polymers [18] and graphene films [19,20]. In recent years, conducting polymers have been also proposed as transparent films to be used in place of metals or oxides [21].
  - Source (S) and drain (D) are often metallic films with appropriate work function to enable efficient charge injection into the organic layer. Large efforts have been devoted to the fabrication of transparent source and drain electrodes to directly collect all the light emitted in the device. Similar approaches as described for the gate electrode have been proposed, although attention should be paid to compatibility of materials and fabrication process, if the electrodes are on the top of the organic material. For configuration requiring bottom contacts (directly on substrates), conventional lithographic methods can be used to achieve µm-scale transistor channel lengths and high resolution.
(b) Organic layer is the active part of the device, where charge transport and light emission occur. This can be either a single layer or a multilayer structure, or a single crystal (see later in the manuscript), where both charge transport and light emission can occur, depending on material properties.
(c) Dielectric layer electrically isolates the gate from the source and drain electrodes needed for the *field-effect* to take place and to be able to induce polarization at the interface to enable transport in the organic active layer. Dielectric thickness smaller than the channel length (at least one order of magnitude) allows *field-effect* transport to occur.

### 2.2. Charge Transport Mechanism and Light Emission in OLET

Emission of light in organic light emitting transistors originates from the efficient radiative recombination of holes and electrons in the active layers (e.g., organic semiconductors) under appropriate bias conditions and, from a more fundamental point of view, offers the possibility of directly visualizing charge distributions and recombination phenomena within the device.

The current flowing between the source and drain electrode (*I_D_*) can be controlled through different biases (electric fields) applied between source and the gate electrodes (*V_G_*) and source and drain (*V_D_*). In most cases, the source electrode is grounded. These electric fields induce an accumulation of charges at the interface between the dielectric and the organic semiconductor, which can transport preferably holes or electrons (*p*-type or *n*-type, respectively) and in some cases both (ambipolar materials).

Let us consider a *field-effect* device based on a *n*-type material (in case of *p*-type, the mechanism remains valid for holes but with inverted polarities).

**Figure 2:**
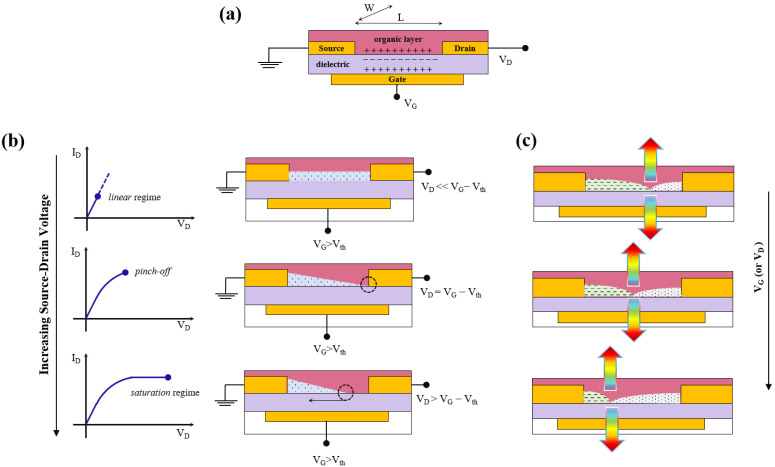
Organic light emitting transistor transport mechanism and light emission. (**a**) Schematic drawing representing the transistor structure and features (*V_D_*: drain-source voltage, *V_G_*: gate voltage, *V_th_*: threshold voltage, *W*: channel width, *L*: channel length). (**b**) Charge transport regimes and corresponding transistor current-voltage curves (top to bottom: linear regime, pinch-off and saturation regime). (**c**) Electrons and holes distributions can be spatially controlled through appropriate bias within the channel.

Increasing the value of *V_G_* (>0) induces a density of electrons accumulating at the interface; however, not all the charges are mobile and will then participate in the conduction, since deep traps will be the ones to be filled first from available charges (Figure 2a). The number of accumulated charges is proportional to *V_G_* and the capacitance of the dielectric layer. We can thus distinguish different regimes, as schematically simplified in Figure 2b:

(a) Linear regime (*V_D_* << *V_G_*), where applying small source-drain biases induces a linear gradient of charge density between the injecting electrode (source) and the extracting electrode (drain). The current between source and drain is given by (Equation (1)):
  (1) $$I_{D,lin}=\frac{W}{L}\mu_{lin}C_{i}\left(V_{G}-V_{th}\right)V_{D}$$
  where *C_i_* is the gate capacitance per unit area, *µ* is the field-effect mobility, *W* and *L* the transistor channel width and length, respectively. In this limit, the linear field-effect mobility can be calculated through the gradient of the transfer curve (*I_D_* vs. *V_G_* sweep, at constant value of *V_D_*) (Equation (2)):
  (2) $$\mu_{lin}=\frac{\partial I_{D,lin}}{\partial V_{G}}\frac{L}{WC_{i}V_{D}}$$
(b) Pinch-off, further increasing *V_D_* leads to the so-called *pinch-off* condition where *V_D_* = *V_G_* − *V_th_*, corresponding to the formation of a depletion zone close to the drain electrode and a space-charged limited current can start to flow across this narrow region. The transistor switches from its OFF state to ON state.
(c) Saturation regime, increasing *V_D_* even further leads to a spatial increase of the depletion area but not an increase in the drain-source constant, which remains constant and it is given by (Equation (3))

(3) $$I_{D,sat}=\frac{W}{2L}\;\mu_{sat}C_{i}{\left(V_{G}-V_{th}\right)}^{2}$$

The *field-effect* mobility and the threshold voltage can be then calculated through the linear fit of the square root of *I_D_*_,*sat*_, according to (Equation (4))

(4) $$\sqrt{I_{D,sat}}=\sqrt{\frac{W}{2L}\mu_{sat}C_{i}}\left(V_{G}-V_{th}\right)$$

For the *field-effect* to occur, the gate dielectric thickness must be at least one order of magnitude smaller that the channel length, condition for which the lateral field from source-drain bias can be neglected. If this condition is not met, a space charge-limited bulk current will prevent the device to reach saturation, and the gate bias will not enable the switching behavior of the device.

When both charges are injected into the active layer, applying appropriate biases allows to spatially tune charges distributions within the channel; at the location where the two distribution fronts meet, exciton can form, radiatively decay and thus emit light with different wavelengths and intensities, as shown in Figure 2c.

### 2.3. Operation Mode: Unipolar vs. Ambipolar

**Figure 3:**
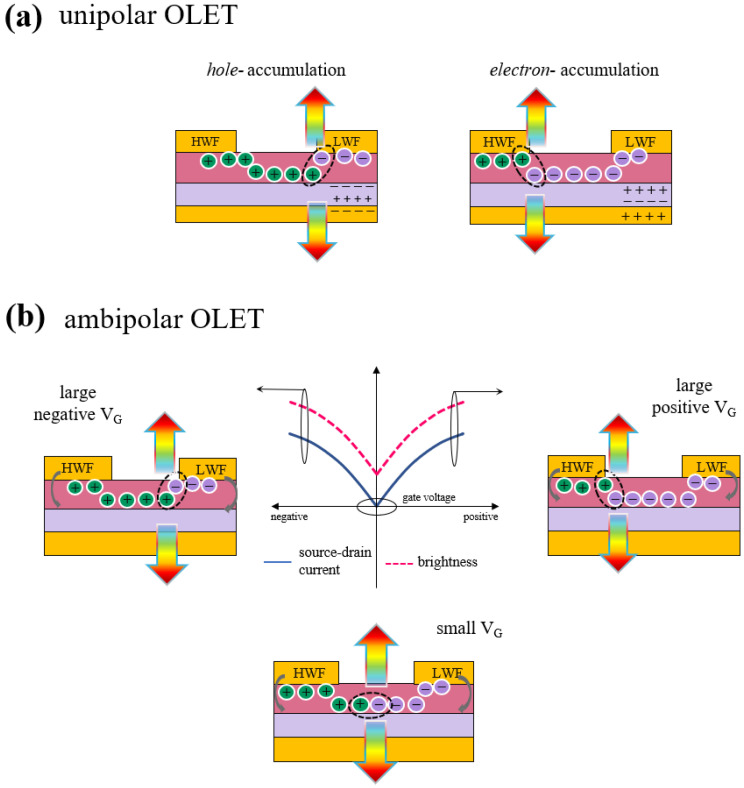
OLET operation: unipolar vs. ambipolar regime. (**a**) Unipolar regime: transport in dominated by holes (electrons), which are injected into the device from the source (drain), but only holes (electrons) accumulate in the channel. (**b**) Ambipolar regime: in the limit of large negative (positive) gate voltage, holes (electrons) accumulate in the channel, and light emission occurs close to the LWF (HWF) electrode (known as *e*(*h*)-injecting electrode). For small gate bias, both charges are injected in the channel, where light emission occurs. Adapted with permission from [22] © (2019) John Wiley and Sons.

Organic light emitting transistors can operate in unipolar mode, if one of the charges, either holes or electrons, is dominating the transport (also known as *p*- or *n*-transport regimes, respectively) or in ambipolar regime, as shown in Figure 3 [22]. In case of unipolar regime with *h*(*e*)-dominated transport, the holes (electrons) are injected into the device from the source (drain), but only holes (electrons) accumulate in the channel (Figure 3a). Light emission then occurs in the proximity of the *e*-injecting electrode with low (high) work function (LWF or HWF), with leads to a stationary light emission being dominated by only one type of charge. In unipolar devices, the emission efficiency is typically low since the exciton radiation and the light extraction are affected by the proximity of the metal contact.

In the case of ambipolar regime, both charges are injected in the organic semiconductor, which is at the same time capable of transporting both holes and electrons that accumulating in the channel, where they can recombine and lead to emission of light (depending on the polarity). Drain-source and gate biases can be used to spatially move the emission zone within the channel. Figure 3b shows transfer characteristics with both electrical and optical output for an ambipolar OLET, in which it is possible to identify three distinct regimes: *hole*-dominated, *electron*-dominated and more strictly speaking ambipolar behavior, which can be seen as a crossover between those two transport regimes [23].

In the limit of large negative (positive) gate voltages, holes (electrons) accumulate in the channel and light emission occurs close to the LWF (HWF) electrode (*e*(*h*)-injecting electrode). In both cases, the charge transport is unbalanced respect to one of the charges which limits the overall efficiency of the device. For small gate voltages (vicinity of zero), both electrons and holes charge density fronts meet within the channel in a balance transport regime, and light emission will occur in the middle of the channel. Although brightness level is rather low, the balance regime between holes and electrons is balanced, thus leading to a more efficient device.

In this condition, the current in the device can be seen as the overlap of both charge transport regimes, with each charge density characterized by its own mobility (*μ_e_* and *μ_h_*) and threshold voltages (*V_th_*_,*e*_ and *V_th_*_,*h*_). In saturation regime, the source-drain current is given by (Equation (5))

(5) $$I_{D,sat}=\frac{WC_{i}}{2L}\left[\mu_{e,sat}{\left(V_{G}-V_{th,e}\right)}^{2}+\mu_{h,sat}\left(V_{D}-{\left(V_{G}-V_{th,h}\right)}^{2}\right)\right]$$

Table 2 summarizes the main parameters characterizing organic light emitting transistor, including both the electrical and optical figure of merits for the device. In addition to mobility and threshold voltage already defined, transistor electrical performances are evaluated through the ON/OFF ratio, defined as ratio between the value of drain-source current when *V_G_* = *V_D_* = maximum bias value and the current measured for *V_G_* = *V_D_* = 0 V. A large ON/OFF ratio is desired since it ensures a clear switching behavior of the transistor with conductance modulation as well as negligible leakage currents.

### 2.4. Device Configurations

**Figure 4:**
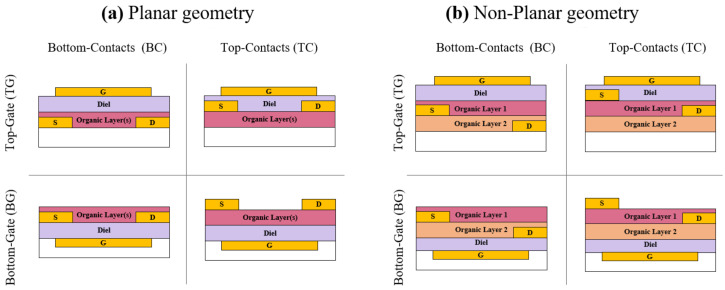
OLET electrodes configuration. Organic light emitting transistor electrodes configurations in case of (**a**) planar and (**b**) non-planar geometry of source and drain. In case of non-planar electrodes and multi-layers stack, source and drain can be located at different level within the device architecture (few representative examples shown).

Organic light emitting transistor can be fabricated in different configurations, depending on where the gate, source and drain electrodes are located within the device, as shown schematically in Figure 4. This becomes of great relevance especially in multilayer structures, where materials and optimized structures can be achieved. In particular, top contacts structures are ideal for charge injection in field effect transistors since a better interface is formed with the underlying semiconductor.

When assessing a suitable structure for organic light emitting transistors, in addition to consideration on the energy of the stack, several other factors should be taken into account:

(a) Fabrication process possibly interfering and/or affecting the organic materials (e.g., electrodes fabrication, dielectric deposition from solution in top-gate configuration);
(b) Organic materials molecular packing and consequently properties, such as mobility, strongly depends on underlying surface;
(c) Materials interfaces are crucial for both charge transport, exciton formation and radiative decay.

When both charges are to be injected, planar geometry for source and drain electrodes is not an optimal configuration, and in fact, one of the charges will always be limited, independently of the polarity and the transistor operating conditions. In the case of multilayer structures, non-planar source and drain can be engineered to optimize injection of each electrodes for the specific charge carriers and also the energetics of each contact with the appropriate semiconductor [24,25].

Electrodes placed in direct contact with the respective charge transport and emissive layer in a multi-layer structure have been shown to lead to a large drop in contact resistance and to facilitate a higher recombination efficiency of electrons and holes, resulting in a substantial enhancement of the brightness up to around 800 cd/m^2^, EQE about 20 times larger than reference sample and ON/OFF ratio of the device larger than 10^5^ [26].

### 2.5. Active Organic Layer

**Figure 5:**
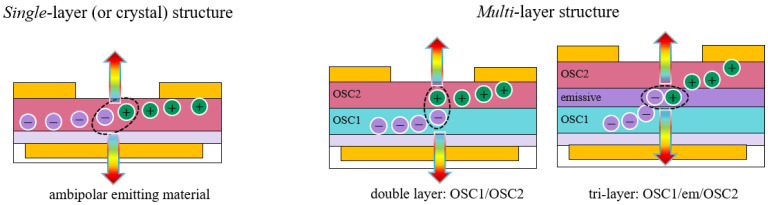
OLET: single vs. multi-layer structure. (**Left**) single-layer (or crystal) organic light emitting transistor, where a single material is responsible for both charge transport and light emission, while (**right**) multi-layer structure includes two or more layers, with each layer being intended for transport and/or light generation.

In organic light emitting transistors, the active layer, where charge transport and light emission occur, can be either a single layer (or single crystal) or a multilayer structure, as shown in Figure 5.

(a) Single layer/single crystal.

First generation organic light emitting transistors have exploited a single organic semiconductor capable of conducting charges and at the same time of emitting light, whether in the form of polymers, single crystals or small molecules.

Hepp et al. first observed in 2003 light emission in a single thin film based on polycrystalline tetracene in a unipolar bottom-gate/bottom contacts OLET structure with interdigitated gold source and drain electrodes on Si/SiO_2_ substrate. Light emission was located at the edge of the drain electrode [27], resulting from different charge injection from the electrodes, with a strong electric field enhancement nearby the drain. A sharp voltage drop occurring at the interface between the dielectric layer and the organic material modifies locally both the HOMO and LUMO (highest-occupied and lowest-unoccupied molecular orbital) of the tetracene molecule at the metal interface, thus favoring electron injection via tunneling process enabled by the non-ohmic contact [28].

Further, large effort has been dedicated to developing organic light emitting transistors based on light emitting polymers (LEPs). Commonly used LEPs are based on F8 (poly(9,9-dioctylfluorene) (e.g., F8BT: poly(9,9-di-*n*octylfluorene-*alt*benzothiadiazole; F8BTBT: poly((9,9-dioctylfluorene)-2,7-diyl-*alt*-[4,7-bis(3-hexylthien-5-yl)-2,1,3-benzothiadiazole]-2′,2″-diyl)), and PPV (poly (para-phenylenevinylene)) copolymers (e.g., SuperYellow, MEH-PPV: Poly[2-methoxy-5-(2′-ethylhexyloxy)-1,4-phenylene vinylene]). Initial studies have been carried out in transistors using either SiO_2_ or polymethylmethacrylate (PMMA) as gate dielectrics, with preference for this latter one given its good dielectric properties, solubility in solvent orthogonal to LEPs and limited amount of residual -OH groups on the surface.

Zamuseil et al. demonstrated in 2006 for the first time ambipolar transport behavior in F8BT-based light emitting transistor, with a characteristic bright green emission peak at around 550 nm. OLET in bottom-contacts/top-gate configuration exhibits a balanced transport between the holes and electrons, with mobility values around 7–9 × 10^−4^ cm^2^/Vs for both charges, threshold voltages of +30 V (electrons) and −20 V (holes) with a maximum EQE of 0.75%, with light located within the channel [29].

A factor of 10 improvement in the efficiency of solution-process F8BT ambipolar light emitting transistor (EQE > 8%) can be achieved by including a thin zinc oxide layer to enhance electron injection from gold electrode into F8BT and a totally reflecting silver gate electrode. Such device exhibited a luminance efficiency larger than 28 cd/A, which is one of the highest reported value for single layer light emitting polymer-based OLET [30].

However, one common drawback of light emitting polymers is their poor charge carrier mobility (<10^−3^ cm^2^/Vs); while this is highly desirable for fast switching devices, often the strong intermolecular π–π interactions quenches luminescence, leading to low brightness levels. Different strategies can be used to overcome these limitations, at the device structure as well as at the molecular and material level. One possible way to improve the device performances is to modify the device geometry, and in particular by increasing the ratio between the channel width and channel length (see Equations (1) and (3)), where common practice is to use interdigitated source and drain electrodes. Most of the early works on OLET research and study have exploited such device geometry; however, depending on applications, this approach might not be the most suitable one or compatible with the device integration in more complex structures. Improvement can be achieved alternatively also through molecular design and device engineering, with the aim of increasing charge mobility and radiative recombination.

Ambipolar behavior in single-layer OLET can be achieved in case of active layer based on a bulk heterojunction. Rost et al. demonstrated that by co-evaporation of PTCDI-C_13_H_27_ (P13) and α-quinquethiophene (α-5T), both known to be high-mobility *hole*- and *electron*-transport semiconductors, it is possible to obtain OLET with mobility of ~10^−3^ cm^2^/Vs (1–2 order(s) of magnitude smaller than in single layer OFET), leading to a narrow area for light emission within the channel [31].

While this approach holds interesting potentials, unfortunately it cannot be of general use; in fact, blending two or more semiconductors materials is a critical process, which should taking into account chemical affinity between molecule and/or polymers, their HOMO and LUMO energy values, grain boundary formation and how all of these can affect *field-effect* transport and light generation processes. Then, fabrication process is even more challenging when blending polymer with a small molecule, and interfaces become a fundamental aspect in all mechanisms. Further, a precise control of the relative amount of each material is also required to optimize overall properties and at the same time to minimize losses due to quenching phenomena.

**Figure 6:**
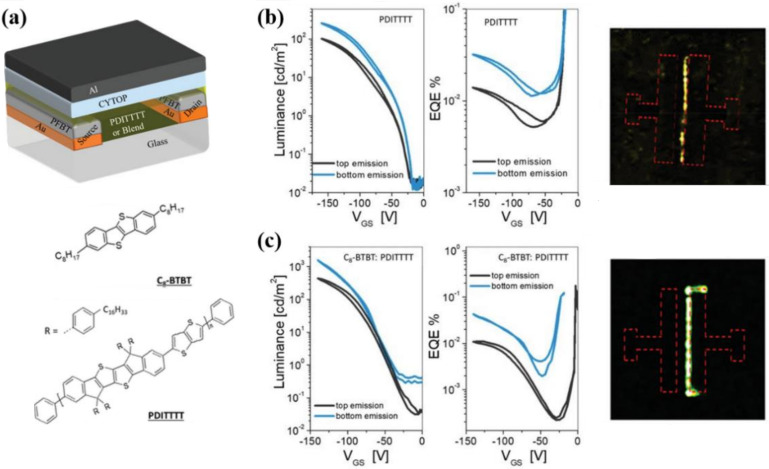
Blended single layer organic light emitting transistor. (**a**) Top gate–bottom contacts OLET based on a single layer blend based on PDITTTT (polymer) and C8–BTBT (small molecule). Luminance and EQE voltage dependence for (**b**) PDITTTT polymer and (**c**) C8-BTBT:PDITTTT blend with corresponding device optical images in ON state. Reproduced from [32], © (2019) John Wiley and Sons.

Recently, Chaudhry et al. have proposed an active layer formed by a blend of a high-mobility small-molecule (C8BTBT) with a polymeric semiconductor poly[6,6,12,12-tetrakis(4-hexadecylphenyl)-6,12-dihydroindeno[1,2-b]indeno[2′,1′:4,5]thieno[2,3-d]thiophene-2,8-diyl]thieno[3,2-b]thiophene-2,5-diyl] (PDITTTT), as schematically shown in Figure 6a. Figure 6b,c suggests that introducing an high-mobility small molecules within the conducting polymer enables a high-mobility single layer light emitting transistor (~3.2 cm^2^/Vs) with improved light output (brightness of 1600 cd/m^2^, EQE = 0.04%), thus representing a potential approach towards single layer OLET high performances device [32].

Growth of organic single crystals plays a key role in the development of high-performances devices, both for organic field-effect as well as for light emitting transistors. Single crystals exhibit limited number of defects (or no defects), which enables high current density and ambipolar behavior and a well-ordered assembly of molecules along preferential directions, often controllable through substrate engineering. On the other hand, the compact molecular structure also creates more nonradiative decay paths for excitons, resulting in low luminescence efficiency. The quality of single crystal can be improved by treating the underlying surface with different self-assembled monolayers (SAMs) such as hexamethyldisilazane (HMDS), phenyltrichlorosilane (PTS) and octadecyltrichlorosilane (OTS), which prevent the formation of cracks and surface non-uniformities due to growth condition (high temperatures). OLET based on single crystals often requires small work function metals (e.g., Ca, Mg) as electron-injecting electrode, and their performances have been improving significantly, while reaching high values of mobility (>10 cm^2^/Vs for rubrene [7]) and opening the way to more precise and detailed studies of the effect of defects and impurities of intrinsic materials properties.

Takenobu et al. reported large current densities in organic light emitting transistors based on single crystals including rubrene (red emission) and tetracene (green emission), both with a well-balanced ambipolar behavior. These devices exhibit large holes (electrons) mobilities of 2.3 (0.27) cm^2^/Vs for tetracene, and 0.82 (0.27) cm^2^/Vs for rubrene, with light emission occurring nearly at *V_G_* = 0.5*V_D_*, with maximum EQE of 0.03% and 0.015%, for tetracene and rubrene, respectively [33].

Thiophene/phenylene co-oligomers (TPCOs) have shown high luminescent quantum efficiency when in single crystal form, along with their versatility in fabrication methods (both in thin films and single crystals) [34]. Kanazawa et al. found for α,ω-bis(biphenylyl)terthiophene (B3PT) crystal a fluorescent quantum efficiency of approximately 80% at room temperature, which is much larger than for example of rubrene and tetracene (~1%) [35]. Depending on growth condition, BP3T-based OLET can show both ambipolar behavior when in amorphous thin film configuration and unipolar behavior when in single-crystal devices, with hole mobilities up to 0.1 cm^2^/Vs [36].

Currently, fabricating high-quality single crystals remains a significant challenge, and it prevents a broad investigation, understanding and thus tuning of their properties. New approaches and strategies are thus needed to enable a large use of single crystals in optoelectronics organic devices.

Yamao et al. have proposed an AC-driven gate signal to promote ambipolar charge transport in single crystal OLET; in fact, the AC sweep enables a gold electrode to inject both electrons and holes. This leads to an efficiency of 3.7 × 10^−4^% in a thiophene/phenylene co-oligomer based OLET [37]. Based on the same approach, light emission was also achieved by Kajiwara et al. in a device based on a bi-layer crystal (one *p*-type, one *n*-type), which favors injection of both holes and electrons leading to a maximum EQE of 0.045%. This approach might potentially represent an interesting tool and approach to achieve bright and efficient light emissions in single-crystal OLETs [38], with values of brightness and EQE that are still very low; further, one should also consider if AC-driving mode is potentially compatible with the targeted applications.

(b) Multi-layer structure.

Given the limited availability of efficient electroluminescent organic small molecules with large mobility values for both holes and electrons [39], a successful strategy for improving light emission performances consists of implementing an OLET structure with multiple layers (Figure 5), which allows the spatial decoupling of the region of charge-carrier density from the one where light emission occurs. This allows the horizontal *field-effect* transport, exciton formation and radiative decay to be independently addressed and optimized.

In case of a bi-layer structure, it can comprise:

- Two organic semiconductors (one *n*- and one *p*-type) with the exciton forming and further decaying at the interface between the two, with charges brought in close proximity through *field-effect*;
- One charge-transport layer and one light-emitting layer, where appropriate bias can be applied to populate the organic semiconductor at the interface with the dielectric layer with majority charges, which start to flow upon application of a drain-source bias. Thus, majority charges are injected into the organic emissive layer where they recombine radiatively with opposite minority charges injected from one of the electrodes leading to light generation. In this structure, the OLET transport is dominated by the nature of the charges present in the OSC.

Preliminary attempts to create smooth bi-layer *p-n* junction from solution-based processes have been recently proposed by Kim et al. which have shown ambipolar transport in a *p-n* heterojunction junction based on both *p*-type and *n*-type conjugated polymers (PDVT-10 and P(NDI2OD-T2), respectively) [40]. Large part of this work has focused on assessing conditions for solvent orthogonality between those two materials; while the bi-layer stack showed ambipolar behavior but no light emission; nevertheless, these are promising findings towards the development of fully solution-processed multi-layer organic light emitting transistor.

Rost et al. demonstrated simultaneous *p*- and *n*-channel formation in a single heterostructure device based on pentacene (*h*-transport) and *N*,*N*′-Ditridecylperylene-3,4,9,10-tetracarboxylic diimide (PTCDI-C_13_H_27_, *e*-transport material), where electrons and holes are injected from Mg top and Au bottom contacts into the PTCDI-C_13_H_27_ and pentacene layers, respectively, to improve device mobility (10^−3^–10^−4^ cm^2^/Vs). This enables efficient carrier injection while choosing a high-work function metal (Au) for hole injection and a low-work function metal (Mg) for electron injection [41].

Namdas et al. used a combination of a *p*-transport layer of poly(2,5-bis(3-alkylthiophene-2-yl) thieno[3,2-b]thiophene) (PBTTT) and light emitting polymer SuperYellow. OLET transport regime is dominated by the *p*-type underlying materials, and it shows brightness level of ~2500 cd/m^2^ with an EQE of 0.15%, which is mainly limited by the limited amount of minority carriers [42]. In a very similar structure, Chaudhry et al. have shown that by controlling the nanoscale morphology of diketopyrrolopyrrole-based semiconducting copolymer (DPP-DTT) deposited underneath a light emitting layer (SuperYellow and PCAN, an anthracene-based molecule for blue emission), it is possible to achieve high current density and mobility values (~7.6 cm^2^/Vs), which enable brightness values as high as 29,000 cd/m^2^ with an EQE of 0.4% for SuperYellow and 9600 cd/m^2^ with an EQE of 0.7% for PCAN [43].

Zambianchi et al. used a very similar bi-layer approach, while exploiting a new material DiPAXA, an anthracene-based material known for its excellent photoluminescence and electroluminescence properties, poorly crystalline film morphology and HOMO-LUMO energy level fine-tunability, to target deep-blue emission in OLET. Deposited in a bottom gate-top contact configuration on top of a high-mobility *p*-type organic semiconductor (C8-BTBT), the bi-layer organic light emitting transistor showed a unipolar charge transport regime with hole mobilities up to 0.32 cm^2^/Vs, a maximum external quantum efficiency of 0.13% and CIE coordinates of (0.18, 0.21), closer to the reference coordinate for blue defined by more common standards (PAL, NTSC) [44].

Namdas et al. showed light emission in a bilayer bottom gate/top contacts Ca/Ag source and drain where the active layer is based on a hole transporting polymer, poly(2,5-bis(3-tetradecylthiophen-2-yl)thieno[3,2-b] thiophene, PBTTT) and Super Yellow, with OLET characterized by peak brightness above 2500 cd/m^2^ and EQE of approximately 0.15% [40].

Bi-layer (and multi-layer) configurations are often based on organic materials deposited by high-vacuum sublimation, since this provides a higher degree of control over morphology, thickness and molecular packing of each materials, in particular at the interface. This is in fact crucial for the development of high-performance organic light emitting transistor since this is the location where most likely excitons are forming. In fact, developing the multilayer active layer in OLETs fully by solution-processed method does not allow for a precise control of packing, thickness and interface; further, chemical and physical affinity between two adjacent materials requires extra effort to investigate, independently of the specific adopted device configuration.

While increasing the complexity of the organic stack, a tri-layer structure allocates emission and field-effect transport to three distinct layers in the active stack, and thus, charge transport and light generation processes can be addressed and further optimized individually. The organic stack consists of three different organic layers (Figure 5): the first and the third layers are field-effect *hole*- and *electron*-transporting semiconductors, respectively, whereas the intermediate layer is where the electron-hole recombination and emission processes take place. Electrons and holes are transport upon *field-effect* to the intermediate layer, where the exciton can be formed and subsequently decays radiatively. For the emissive later, host-guest systems are often chosen since they favor either charge transfer or energy transfer, thus leading efficient radiative recombination.

Capelli et al. demonstrated for the first time in 2010 that such multilayer OLET structure can potentially outperform OLED, when using the same set of materials with maximum reported EQE of 5% and independently of the material sequence within the stack [8]. While from energy considerations the stacks behave in the same way, it is important to note that structure, molecular packing and interfaces might be different, depending on fabrication sequence (for more details, we refer the Reader to [45]).

Introducing additional layers to optimize one or more of the mechanisms occurring in the device might be also possible, as it often already happens in organic light emitting diodes, where for example injection and/or blocking or doping layers and blended approaches can also be introduced to improve device performances. Patterning one or more of these layers has been demonstrated to be also a viable way to control even further the location of the recombination zone [46]. Nevertheless, one should also consider that the higher the number of interfaces in the device, the more challenging it becomes to control and tune the device properties, while maintaining similar optical and electronic performances. This is relevant for both vacuum- and solution-processed materials, whether they are semiconductors or emissive materials.

In case of multi-layer, one interesting approach holding great potential is the so-called *hybrid stack*, in which the active layer is based on a combination of both organic and inorganic materials; in many cases, metal oxide semiconductors are used in place of the OSCs due to their high-mobility values.

Walker et al. reported a hybrid OLET, with the active layer based on a high-mobility, solution-processed cadmium sulfide layer (in direct contact with the dielectric layer) and polymer Super Yellow in a non-planar source/drain device geometry. Such structure exhibits electron mobilities of the order of 20 cm^2^/Vs, ON/OFF ratios >10^7^ and external quantum efficiency of 0.02% at 2100 cd/m^2^ [47].

Similarly, Park et al. uses zinc-oxynitride (ZnON) as the inorganic semiconductor transport layer given its very high electron mobility (*µ_e_* > 120 cm^2^/Vs) and high optical transmittance (>87%) [48], again in combination with Super Yellow. OLET configuration based on bottom-gate and MoO_x_/Au top contacts is characterized by very low threshold (<5 V) despite the use of SiO_2_ as gate dielectric, high brightness of approximately 30,000 cd/m^2^ and a corresponding EQE of 0.10% [49].

Hybrid *n*-type light emitting transistor based on a heterojunction In_2_O_3_/ZnO and Super Yellow has shown low-bias operating condition (<10 V), high electron mobility (~22 cm^2^/Vs), ON/OFF ratio of ~10^3^ and an external quantum efficiency value of 0.02% (at brightness level of 700 cd/m^2^) [50]. Zinc tin oxide (ZTO) [51], indium zinc oxide (IZO) [52], aluminum-doped zinc-oxide (AZO) [53] or oxides heterostructure (In_2_O_3_/ZnO and In_2_O_3_/Ga_2_O_3_/ZnO) [54] have been proposed for high-performance OLETs. The high brightness coupled with a high ON/OFF ratio and low-cost solution processing renders these hybrid devices attractive from a manufacturing perspective and application point of view [45]. Some of these approaches also open the way to the development of all-solution processed light emitting device, which is surely of interest for the development and implementation of low-cost and high-yield manufacturing processes.

Further, using metal oxides as charge transport layers in hybrid OLET configuration allows to overcome one fundamental limitation in terms of organic materials, namely the limited availability of high-performing *n*-type materials, currently representing a major bottleneck towards the achievement of both high-performing OFET and balanced (from a transport point of view) high-efficient organic light emitting devices. In particular, the bi-layer stack approach offers a broad flexibility given the number of both *p*- and *n*-type metal oxides currently available and the versatility in fabrication processes (vacuum and solution-process), potentially fully compatible with OLET architecture. In fact, metal oxides are insoluble to the solvents often used for organic materials and polymer, thus simplifying multi-layer device fabrication. Further, electrical stability under stress bias conditions and environmental stability are also expected to reflect in the overall reliability and robustness of the device, which is an aspect of fundamental importance in the field of organic electronics.

### 2.6. Vertical-Organic Light Emitting Transistor (v-OLET)

A novel concept of vertical-organic light emitting transistor has been recently introduced in the field of organic (light emitting) devices. The *v*-OLET consists of a vertically arranged gate, source, and drain electrodes, where and OLED structure is stacked on top of a capacitive cell. Applying a gate bias induces hole accumulation at the dielectric/organic layer interface, which reduces the Schottky barrier, thus enabling effective hole injection and electron/hole recombination to produce light emission when a negative *V_D_* bias is applied. To allow the electric field (induced by *V_G_*) to be applied to the active layers, the source electrode needs to be either ultrathin or perforated such as carbon nanotubes network, holey graphene or porous ITO, which are often used. Further, the source electrode should also be transparent in the appropriate range of wavelength to allow the extraction of light.

Strictly speaking, this is not a *field-effect* transistor in the more conventional sense since no lateral charge transport and channel conductance modulation is achieved; nevertheless, such vertical structure shows a certain degree of *gate*-like modulation. *v*-OLETs are characterized by a very short channel length (corresponding to the thickness of the active layer, typically of the order of several 10 s of nm), and they are compatible with integration in a vertical structure. Such structure holds potentials as light source to increase resolution for example in array of devices, as for example in emissive display.

## 3. Gate Dielectric for High-Performance Organic Light-Emitting Transistors

Achieving high-performance organic light emitting transistors requires reaching large drain current at low biases and large light output; this can be obtained in several ways, while targeting one or more building blocks of the transistor itself:

(a) High-mobility and high-performance organic semiconductors [55,56,57];
(b) Luminescent materials, with high fluorescent/phosphorescence yield in solid state and tunability of color coordinates [58];
(c) Dielectric layer (capacitance and interface with active layer) [59];
(d) Interfacial and bulk trap states at different boundaries within the device [60].

Among those, we here focus on the role played by the dielectric layer and how it is possible to engineer it in order to improve OLET properties.

Dielectrics are materials capable of inhibiting charge transport within the layer itself. When applying an electric field, a shift in charge distribution is induced in the layer, leading to polarization effect. In more conventional description, the application of a bias between two electrodes separated by a distance *d* induces an electric field E(=Vd) with a charge per unit area given by Q=ε0E=ε0Vd, from according to which it is possible to define the capacitance per unit area Ci=QV=ε0d.

If a dielectric material is introduced between the two electrodes, the capacitance value is increased by a factor *k*, known as the dielectric constant and which is a characteristic of the material. As a result of the materials polarization, the value of the capacitance per unit area can be written as Ci=ϵ0kd.

Another important parameter is assessing dielectric properties of a materials is the maximum electric displacement (*D_max_*) that the dielectric layer can sustain (before breaking and losing its insulating behavior), given by $D_{max}=\varepsilon_{0}kE_{B}$, with *E_B_* (=*V_B_*/*d*) being the breakdown field. For fields larger than *E_B_*, the dielectric layer becomes conducting and it does not act anymore as dielectric layer.

Gate dielectric plays a fundamental role in the development and improvement of device performances in organic thin film transistor and therefore in organic light emitting transistors.

In saturation regime, OLET drain-source current *I_D_* is proportional to $\frac{W}{L}\mu C_{i}$ (see Equation (3)); thus, few approaches can be used to enhance this current, such as using high-mobility semiconductors and increasing the *W*/*L* ratio or the value of *C_i_*. To increase the value of the capacitance, one can either reduce the thickness of the dielectric films ($C_{i}\propto k/d)$ or use a dielectric characterized by a high value of the dielectric permittivity (high-*k*). The first one, although quite viable, might not ensure pinhole-free and smooth dielectric films.

As a general approach, dielectric materials should have a large dielectric constant and be processable into *defect*- and *pinhole*-free high-quality films to enable low-bias driven transistors, with large ON/OFF ratio and fast switching speed. Many of the preliminary works in the study of organic light emitting transistors have used either low-*k* dielectrics (oxides such as SiO_2_ and polymers like PMMA, CYTOP or PVA), all widely known materials in the scientific community but which inevitably lead to high operating voltage (>tens of Volt), which represents a great limitation for the potential applications.

We refer the Reader to some interesting and more detailed and comprehensive review on gate dielectrics relevant for the field of transistors [12,61]. We will here just summarize few main concepts and we will highlight the role of the dielectric to achieve high-performance organic light emitting transistors.

Although increasing the value of the dielectric permittivity of the gate dielectric enables the increase of the charge carrier mobilities and low-voltage operation [62], one should also pay attention to the effect that a highly polarizable dielectric can have on the organic layer; in fact, strong charge-dipole coupling in high-*k* dielectrics, including oxides, can lead to charge carrier localization and polarons formation [63], both leading to detrimental change of mobilities and metal-insulator transition depending on the permittivity. This effect is negligible in polymer dielectrics with low dielectric permittivity (*k* < 4), where dipole moments are randomly oriented within the gate dielectrics [64].

### 3.1. Interface between Dielectric and Organic Semiconductors

The interface between the dielectric layer and the organic materials plays a crucial role in the operation and affects the performances of the transistor [58], since this is the interface where charge accumulation and transport primarily occur.

In the limit of bottom-gate configurations, physical and chemical surface properties of the dielectric influence both the structural and energetic disorder of the organic material. In fact, surface chemistry and texture/structure can influence diffusion, aggregation and crystallization of the organic materials, leading to a variation in its molecular order and thin-film morphology, which strongly affect transport properties [65,66]. Grafting self-assembly monolayer (SAM) onto the dielectric surface is an effective tool to modify the surface tension to control molecular orientation [67], grain size and boundary density [68] and polymorphism [69,70] in both vacuum-sublimed and solution-processed organic material, with an overall improvement of the device performance [71]. Smooth and defect-*free* dielectrics often lead to large grain sizes and reduced grain boundaries, thus resulting in a low trap density and increased mobility [72,73]. Further, nanostructured dielectric surface can also “direct” to an improved alignment of polymer films with improvement of the charge transport [74,75].

If dielectric is used in a top-gate configuration, additional factors must be considered, including compatibility of the fabrication process with underlying organic materials and possible interaction of the solvent contained, if dielectric is solution-processed [76].

Water molecules physisorbed on the surface of the dielectric, hydroxyl groups (in case of oxides like SiO_2_) and polar SAMs carrying *e*-withdrawing (e.g., fluorine) or *e*-donating (e.g., NH_2_) groups can act as sites for charge trapping, thus limiting the charge transport [77,78]. Using hydrophobic SAMs can be effective in electrochemically passivating these trap sites, while preventing physisorption of water or similar species and improving bias-stress stability [79]. Surface treatment such as oxygen plasma can also drastically reduce charge traps density at the dielectric interface [80]. On the other hand, polymeric films [81] and non-polar SAMs can also minimize those effects, thus improving transport properties and even enabling ambipolar behavior [82]. When using polymers such as poly(vinyl alcohol) [83], poly(styrenesulfonic acid) [84] and poly(vinylphenol) [85], one should also consider possible ion migration caused by water doping/absorption following humidity conditions, where this mechanism can induce a shift of the transistor threshold voltage(s). PMMA and CYTOP are for example preferred materials to avoid ion migration [86].

The organic/dielectric interface can be also potentially engineered to introduce additional functionalities. Zhang et al. have demonstrated that using a photosensitive SAMs (SP, spyropyran), sandwiched between the oxide (SiO_2_) and the organic semiconductor, enables to control the transistor electrical properties though external illumination; in fact, light (with different wavelengths) induces photoisomerization, which modifies the local electric fields at the interface leading to conduction channel modulation and threshold voltages variation [87].

### 3.2. High-k Inorganic Dielectric Materials (Oxides, Nitrides, etc.)

Silicon dioxide (*k*~3.9) has been extensively used as dielectric layer for decades now, in the silicon-based microelectronics industry, for which a very high quality, smooth and thin layer can be routinely fabricated by thermal oxidation directly on Si wafer. This has enabled high reproducibility over the same wafer and over multiple wafer batches. Following the continuous miniaturization of ICs (*nm*-scale node for ICs), silicon dioxide has now reached its intrinsic physical limits [88]; in fact, a 3 nm SiO_2_-based capacitor exhibits a leakage current level of 10^−5^ A/cm^2^ when applying 1 V, which becomes 10^6^ larger when reducing just by half the dielectric thickness. Thus, *nm*-scale thin silicon oxides layer cannot anymore ensure a well performing insulating behavior in the device.

Thus, new dielectric materials with higher values of *k* are indeed required to ensure reliable dielectric properties and high performances transistors, both organic and inorganic. From a device perspective, reducing driving voltages has the key advantage of enabling low-power consumption.

**Figure 7:**
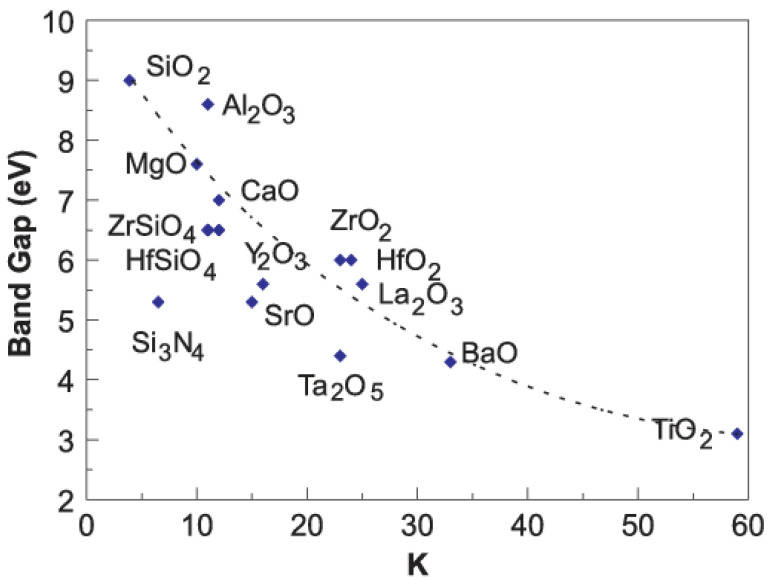
Dielectric permittivity of inorganic materials. Bandgap as a function of the dielectric permittivity for different inorganic dielectrics, mainly oxides. Reproduced from [90], © (2004) EDP Sciences).

Figure 7 shows the values of dielectric permittivity for a broad collection of inorganic materials (mostly oxides) which holds potentials to replace and go beyond SiO_2_ [89]. Based on Equation (3) and in the limit of same geometry, doubling the value of dielectric constant (e.g., changing dielectric from SiO_2_ to Al_2_O_3_, for example) can lead to a two-fold increase in the value of the saturation current, thus improving also the values of mobility and threshold voltage. While those considerations are of general applicability to any transistor-based platform, additional requirements might be needed in the case of OLET, considering the presence of the light in the device. One very common requirement is the dielectric layer to be optically transparent if the light is extracted also through the gate electrode.

High-*k* dielectrics hold then great potential for the development of high-performance organic (light emitting) transistors; nonetheless, the interface between the organic layer and inorganic dielectrics presents a large number of hydroxyl groups which act as defect and trapping sites, detrimental for device performance and leading for example with hysteresis behavior, poor stability and low charge mobility. Different passivation methods are proposed and successfully implemented to overcome this limitation [90,91,92].

Chaudhry et al. have recently shown a very interesting approach based on a bi-layer dielectric structure composed of SiN_x_/SiO_2_ (200 nm/400 nm) (SiN_x_ having a dielectric constant of 9–10). Although the use of SiO_2_ in a vertical stack reduces the total capacitance, the work shows that nanostructuring the nitride layer to create a *groove*-like surface leads to the formation of dense highly aligned nanofibers of the organic materials (DPP-DTT) deposited on top, which enables a more efficient field-effect transport and injection process. The dielectric surface has been also functionalized to decrease trap states. While the nanostructure induces only a small change of the overall stack capacitance (from 5.2 nF/cm^2^ for flat SiN_x_ to 5.6 nF/cm^2^ for grooved surface), very promising results are obtained for two different emissive materials (Super Yellow: *µ* = 7.6 cm^2^/Vs, ON/OFF > 10^6^, brightness ~30,000 cd/m^2^, EQE = 0.4%; PCAN: *µ* = 4.8 cm^2^/Vs, ON/OFF >10^5^, brightness ~10,000 cd/m^2^, EQE = 0.7%) [41]. This approach, while very promising, is in principle strongly dependent on the nature of the interaction between the dielectric surface and the first organic layers deposited, and thus, it cannot be of general use.

In 2016, Soldano et al. showed that integrating Al_2_O_3_ thin film grown by vacuum-based atomic layer deposition (ALD) to drastically reduce the threshold voltages and the overall operating bias range while maintaining comparable optical signal showed large improvement when compared to PMMA benchmark devices. In particular, ALD enables the deposition of highly conformal, defect-free thin films at relatively low temperature, with high resistivity and good barrier properties [93,94]. ALD process ensures thickness uniformity overlarge areas, control of film thickness and composition at the atomic level, and compatibility with various substrates and with irregular shapes. Al_2_O_3_ topmost surface is covered with a thin layer of PMMA. Engineering Al_2_O_3_ oxide layer (<50 nm) allows to achieve OLET threshold voltages below 10 V and tune its optoelectronic properties, while maintaining robustness and very low leakage current [95].

Anodized Al_2_O_3_ can be also used effectively to fabricate bottom-gate/top (non-planar) contacts OLET with very low threshold voltage (<5 V). A thin layer of polystyrene coating to passivate the dielectric surface and non-planar electrodes optimized for hole and electron injection into respective organic semiconductors leads to high value of hole mobility in saturation regime (>2 cm^2^/Vs) with threshold voltage at ~1 V with ON/OFF value of ~4 × 10^5^ [96].

Chaudhry et al. proposed that dielectric oxide layers produced by solution-process method, consisting of a vertical stack of a thin layer of Al_2_O_3_ (following oxidation of Al gate electrode) and ZrO_x_ deposited by spin coating, starting from zirconium acetylacetonate (Zr(C_5_H_7_O_2_)_4_) precursor and ethanolamine, lead to a *n*-type driven hybrid OLET with threshold voltages below 10 V and high mobilities [48]. Efforts in this direction are very important since solution-based processes are potentially enabling cheap and low-cost production, as well as being fully compatible with flexible substrates.

Low-bias (<10 V) OLET can be obtained using HfO_x_ (*k* ~ 12–23 depending on processing parameters) spin-coated from solution and passivated with a *n*-dodecylphosphonic acid (PA-C12)-SAM, which renders the topmost dielectric surface highly hydrophobic and facilitates the formation of the organic film [97]. Passivating the surface reduces the capacitance per unit area (from ~200 nF/cm^2^ to 160–170 nF/cm^2^), on the other hand ensure lower leakage current in the dielectric layer (~10^−6^ A/cm^2^) [98].

High-*k* oxides have also been successfully implemented in vertical-OLET architectures, with Al_2_O_3_ and HfO_2_ being the most widely exploited and more recently in novel organic permeable base transistor (where the switching behavior is enabled by a permeable base electrode located in the middle of the device structure) [99].

**Figure 8:**
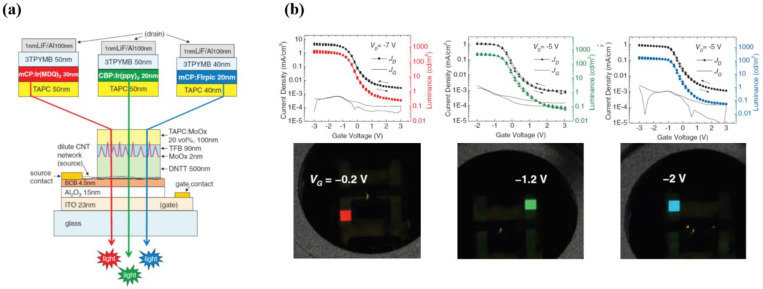
Low–voltage red, green and blue *v*-OLET. (**a**) Schematics of red, green and blue *v*-OLET, using carbon nanotubes (CNTs) as high mobility charge transport layer and corresponding (**b**) transfer curves and optical images for each of the three representative colors. Reproduced from [101], © (2011) The American Association for the Advancement of Science.

McCarthy et al. demonstrated that a thin layer of ALD-grown Al_2_O_3_ (15 nm) capped with a benzocyclobutene (BCB, 4.5 nm) dielectric layer combined with high mobility carbon nanotube (CNT)-based transporting layer can significantly reduce the OLET threshold voltage (as low as ~2–3 V), thus obtaining highly efficient devices for all three different colors (red, green, blue), as shown in Figure 8 [100].

Lee et al. proposed the concept of “overlapping gate” applied to organic light-emitting transistor, consisting of introducing two partially overlapping gates in the device, separated by Al_2_O_3_. This allows to control individual charge carriers in their corresponding layers, which can then be independently injected into the emissive layer. Such configuration leads to high-performance light emitting transistor with high luminance (2190 cd/m^2^) and high efficiency (5.7%).

### 3.3. High-k Polymer Dielectrics

Polymer dielectrics have been widely used in OFET and OLET applications, for mainly two reasons: on one hand, they are characterized by the absence (or very limited number) of traps at the interface which allows for better performing devices, and on the other hand, they enable the fabrication and the development of devices on flexible substrates, key feature relevant for applications in wearable and conformable electronics. When implementing polymer dielectrics, one should consider the following:

- Process compatibility, since fabrication process might require high temperature (annealing) processes, which can affect substrate and the active organic layer (depending on configuration).
-*OH* groups on the surface, which often require a passivation layer at the OSC-dielectric interface, thereby exhibiting inferior properties of mobility, stability and leakage current.

Using polymer dielectrics, even in the case of low-*k* materials, generally ensures good mechanical properties and simple fabrication processes; nevertheless, large thicknesses (>300 nm) are still required to minimize the leakage current, which leads to high operating biases.

Initial studies and development of organic light emitting transistors has seen the extensive use of PMMA as dielectric layer. Zaumsetil et al., for example, have demonstrated F8BT-based organic light emitting transistors fabricated by spin-coating PMMA dielectric in top-gate configuration, showing a well-balanced charge transport for both holes and electrons with saturation mobilities of ~10^−3^ cm^2^/Vs and a quite narrow light emission in saturation regime, at a bias of |100| V [101].

Use of PMMA allows for smooth surface and limited number of traps sites at the interface, thus leading to generally good device performance, with reduced leakage currents and limited hysteresis. For this reason, PMMA is also a material of choice for passivation. On the other hand, PMMA has a value of the dielectric permittivity of 3–4, which prevents device low-bias operation. Thus, search for higher-*k* dielectric is needed to improve device performances and achieve operating condition below 20 V.

Naber et al. presented a top-gate/bottom-contacts OLET based on F8BT, which exploits as a dielectric layer a combination of a high-*k* material (P(VDF-TrFE) with a dielectric constant of ~14) and low-*k* dielectric (polycyclohexylethylene, PCHE with a dielectric permittivity of 2.3). While the high-*k* component allows the low-driving operation condition for the device, the PCHE in direct contact with the light emitting polymer leads to a better interface and helps screening the ferroelectric behavior of PVDF. Although the presence of PCHE reduces the overall dielectric capacitance (11–12 nF/cm^2^), the light emitting transistor exhibits hole and electron mobility of ~0.02 cm^2^/Vs, threshold voltages of around 40 V with a maximum efficiency of 0.75% [102].

**Figure 9:**
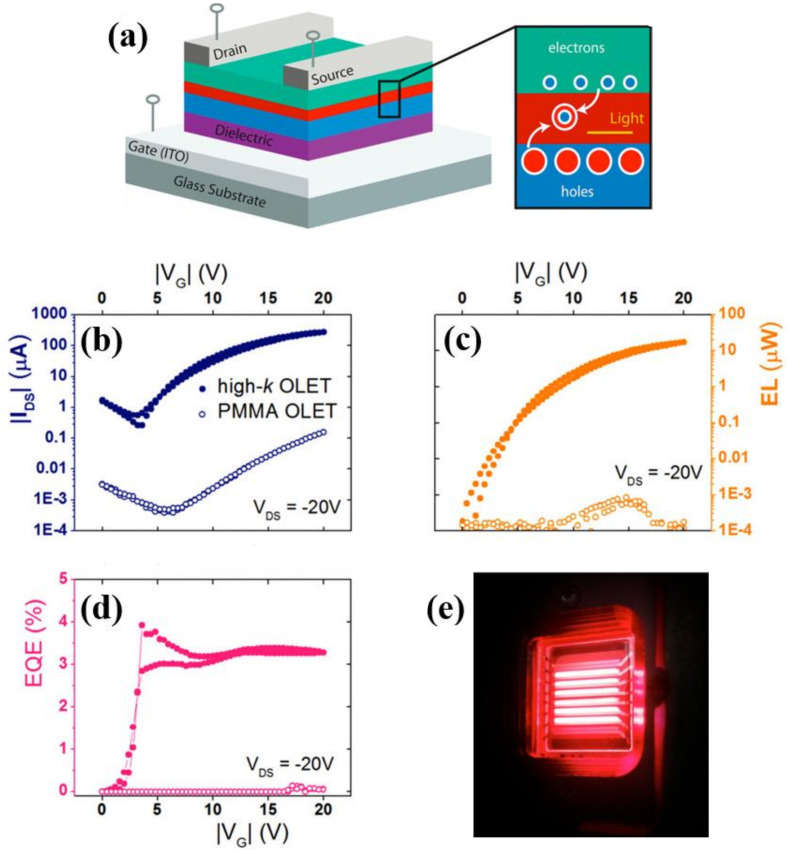
Organic light emitting transistor using high-*k* polymer dielectric layer. (**a**) Schematic representation of the tri-layer OLET, where the dielectric layer is either PMMA or high-*k* polymer P(VDF-TrFE-CFE). (**b**) Drain-source current, (**c**) electroluminescence and (**d**) EQE curves of PMMA- and high-*k* OLETs in the limit of *V_D_* = −20 V. (**e**) Optical image of the red OLET in its ON state. Reproduced from [104], © (2017) American Chemical Society).

In 2017 Soldano et al. proposed a tri-layer bottom-gate/top-contacts OLET using a thick transparent P(VDF-TrFE-CFE) layer as gate insulator, as shown in Figure 9. As compared to Nabel et al. the terpolymer exhibits even a higher dielectric constant value (~27–30). When directly compared to PMMA counterpart (driven at 100 V), the high-*k* OLET exhibits very similar values of saturation currents and charge mobilities (holes: 0.4–0.7 cm^2^/Vs, electrons: 4–8 × 10^−3^ cm^2^/Vs), which however can be reached at much lower biases with threshold voltages of the order of *|*10 V*|* (PMMA, *V_th_^p^* = −50 V and *V_th_^n^* = 30 V approximately), while keeping similar efficiency (~3%) [103].

Very similar work has been shown recently by Nam et al., who have reported a highly efficient and stable solution-processed bottom-gate/non-planar top-contacts organic light-emitting transistor based on P(VDF-TrFE-CTFE) dielectric and an active layer based on a polymer heterostructure including (poly[4-(4,4-dihexadecyl-4H-cyclopenta[1,2-b:5,4-b′]dithiophen-2-yl)-alt-[1,2,5]thiadiazolo[3,4-c]pyridine] (PCDTPT) as charge-transport layer and Super Yellow as the light-emitting component. Such OLET exhibits large drain-source currents (~2 mA) and driving bias below 35 V, with an external quantum efficiency of ≈0.88% (at 2000 cd/m^2^), excellent shelf-life and operational stability. One main difference compared to Soldano et al. is that the device is fully fabricated with solution-processed materials, which thus represents a significant step forward in the quest for flexible, cheap and large-scale fabrication processes for the OLET-based technology platform [104].

Cross-linked polymers have been shown to have smoother surfaces, high electrical field strength, larger dielectric constant, high purity, low leakage current (~10^−8^ A/cm^2^) and high surface hydrophobicity [105]. Further, they are also compatible with Si, ITO and Al gates and with several *p*-type and *n*-type organic semiconductors, and they are potentially *pinhole*-free.

**Figure 10:**
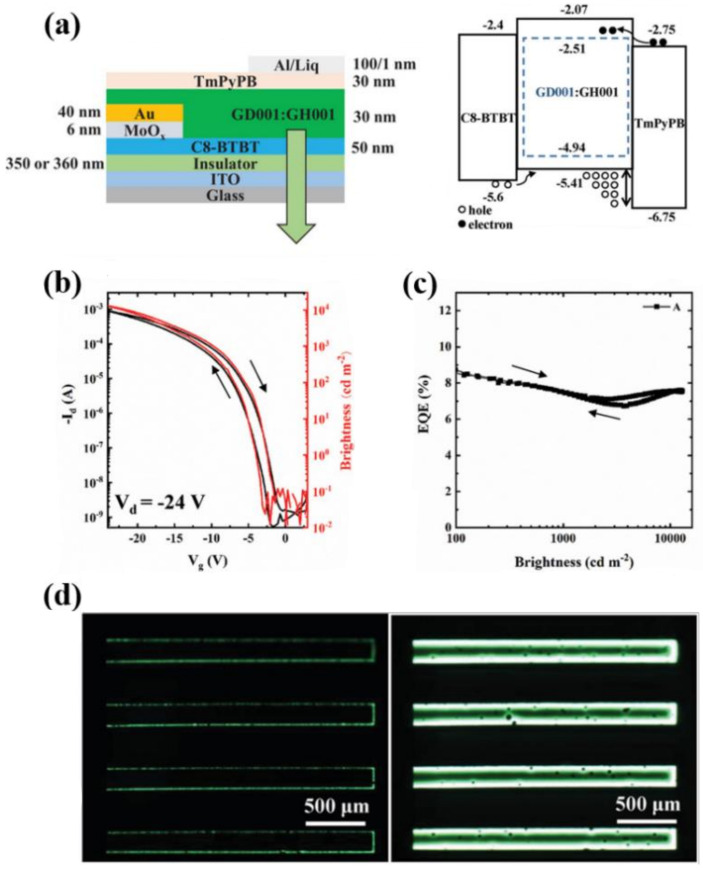
High-performance OLET using cross-linked polymer dielectric. (**a**) Device architecture and corresponding (**b**) energy level, including the schematics for the charge carrier transport mechanism. (**b**) Saturation transfer curve and (**c**) EQE for device in (**a**). (**d**) Optical images of light emission in C-PVA OLET in the limit of (**left**) low- and (**right**) high-current. Reproduced from [106], © (2020) John Wiley and Sons.

Chen et al. have recently shown a multi-layer (three layers) organic light emitting transistor using high-*k* crosslinked poly(vinyl alcohol) (C-PVA) as gate insulator. While combining a *hole*-transport layer with high-mobility, a high efficiency of guest-host system and an optimized non-planar electrodes injection configuration and materials, it was possible to achieve high brightness levels (14,500 cd/m^2^) and an unprecedented external quantum efficiency of almost 9% in inert atmosphere, as shown in Figure 10. One interesting outcome of this work is that the addition of an extra layer of perfluoro(1-butenyl vinyl ether) polymer (CYTOP) on top of PVA enhances the hydrophobic nature of this layer and enables the device to operate in ambient conditions (relative humidity RH = 70%), while retaining very good electro-optical properties (brightness of 13,400 cd/m^2^, EQE > 7%). When this structure is transferred onto flexible substrate, OLET mainly retains its characteristics (brightness of 8300 cd/m^2^, peak EQE of 9%), thus confirming the potentials of the OLET technology platform for flexible applications [106].

Yumusak et al. used PVA in an organic electrochemical light emitting transistor, a special class or organic transistor based on ion migration and where the role of the dielectric is acted by an electrolyte. In particular, PVA as dielectric materials in conjunction with light emitting polymer MDMO-PPV (poly[2-methoxy-5-(3′,7′3′,7′-dimethyloctyloxy)-1,4-phenylenevinylene]) lead to a *p*-driven OLET with clear saturation behavior, with calculated mobility of 2.2 × 10^−4^ cm^2^/Vs [107]. Performances in such devices are far from being optimized ye and further, its general concept applicability might be limited since there is a need of a robust and reliable interaction between organic materials and the chosen electrolyte. Nonetheless, achieving high-performing organic electrochemical light emitting transistors in the future surely represents a fundamental step which will extend the exploitation and development of OLET device platform in fields such as bio-electronics and bio-sensing, where the OLET will bring a light component, which can now be also used in the interaction for example with biological sample.

As expected, using high-*k* dielectric will improve electrical performances, and correspondingly the optical output in organic light emitting transistors; however, several issues still remain and need to be addressed within the context of the specific device platform, including:

(a) Process compatibility, where some fabrication methods might not be (fully) compatible with organic semiconductor and/or plastic-like substrates. Also, several solution-based fabrication methods, will still require additional surface modification to favour semiconductor growth. In addition, in top-gate configuration, all these processes need not to interfere with the already existing organic material;
(b) Mechanical flexibility in the case of inorganic dielectrics, which poses limitation to their use in flexible electronics. On the other hand, using polymer dielectrics for example allows for fabrication of all-printed flexible organic devices. However, polymers are generally characterized by low dielectric constants, thus requiring large thicknesses as gate dielectrics to reduce leakage currents. Few high-*k* polymers are currently available, where the dielectric permittivity values result from high dipole polarizability, which should be taken into consideration in the case of organic light emitting devices;
(c) Affinity with organic materials, where the interfaces are playing a fundamental role in the device operation. For example, passivation layers (e.g., SAMs) have been successfully shown to reduce interfacial traps at this interface and cross-linked polymer dielectrics allows for the deposition of the subsequent layers by solution-based methods, without dissolving the underlying gate dielectric layer.

## 4. Engineering Dielectric Layer for Light Manipulation in Organic Light Emitting Transistors

Gate dielectrics are a key building block to achieve high-performance organic light emitting transistors; their role is not only limited to achieving low-bias, low-power operating condition through the use of high-*k* dielectrics but it can also be exploited to manipulate light, and perhaps enhancing the light output of the device itself.

One major limiting factor in organic light emitting devices resides in the light loss due to waveguide and surface plasmon modes [108] and for which several solutions have been proposed including planar microcavities [109], lens sheets [110], photonic crystals [111], index-engineered substrates [112]. Fabricating cavities in organic thin film devices [113] is not always compatible with device structure and integration in more complex architectures.

For the dielectric to play an active role in device light manipulation, one necessary condition is that the emitted light must pass through and/or interact with the dielectric layer, thus it is required to be optically active at the light wavelength of interest. Manipulating light can include for example change of the characteristic emission peak wavelength, variation of the light output power, suppression or enhancement of specific frequency components, and more.

In this part of the Review, several approaches are presented, in most cases not yet applied to organic light emitting transistors, which holds great potentials in introducing additional functionalities to the dielectric layer.

### 4.1. Quantum Dots Composites Dielectric

With advances in the field of nanotechnology, quantum dots have become an interesting class of nanoscale objects, the optical properties of which can be tailored to lead to emission in different region of the VIS spectrum. Quantum dots are semiconducting nanoparticles (2–10 nm) with electronic properties intermediate between discrete molecules and those of semiconductor bulk, mainly arising from the high *surface-to-volume* ratio. Changing materials and crystallite size allow for tuning the quantum dots photo- and electroluminescence properties within the visible spectrum. Most available work in literature is related to the use of QDs as light emitting centers; one can also exploit luminescent properties of QDs such that the light emitted by the device can indeed stimulate a secondary emission in the QDs.

Grinolds et al. have demonstrated dielectric films made entirely of PbS QDs (in combination with high-*k* oxide HfO_2_), where film properties can be adjusted thorough nanoparticles size and QDs volume fraction, leading to a tunable absorption-photoluminescence spectrum [114]. In this sense, while QDs do not strongly affect the dielectric properties of the layer, they can introduce additional optical emission (component) upon light excitation coming from the OLET primary emission. It is expected that the insertion of the QDs layer do not interfere with the properties of the hafnia layer, which can remain the main dielectric component in a QDs-based OLET.

Although QDs exhibit large quantum yield in solution, their efficiency in solid state the efficiency is strongly reduced due to clustering and agglomeration, which quenches the photoluminescence. Dispersing QDs in a host matrix such as a polymer, can lead to optically active composite structures, which on one hand are largely expected to retain the dielectric properties for the polymer itself, and on the other, are capable of manipulating light due to the present of semiconductors nanoparticles. Further, it is important to recall that such types of composites are suitable for fabrication of devices on flexible substrates.

QDs-based polymer composites can be obtained through: (a) through chemical forces by grafting the polymer onto the QDs or replacing ligands [115], which might require complex synthesis procedure or (b) no chemical force, easier in fabrication but often resulting in low quantum yield (QY) [116].

In the early 2000, Lee et al. have demonstrated a PL quantum yield in a polymer matrix film based on (polylaurylmethacrylate) with CdSe(ZnS) quantum dots of approximately 40%, which is lower than that in solution (49%), but which still hold promises to implementation of QDs based composites as dielectric layer [117]. By tuning the size of the QDs and stabilizing them within the polymer matrix with tri-*n*-octylphosphine (TOP) ligands, the authors demonstrated a broad range of pure and bright mixed-colors (based on engineered mixture of QDs within the same matrix).

Kong et al. recently demonstrated QDs-PDMS composite through in-situ hydrosilylation to produce red (CdSe/CdS), green (CdS@ZnS/ZnS) and blue (CdS/ZnS) light emitting composites with relatively high QY (in the case of green the composites QY indeed results slightly higher than in solution), thus suggesting the possibility of using this class of composites as potential optically active dielectric [118]. Although dielectric permittivity is still quite low in the case of PDMS, it is indeed interesting also that PDMS is often also used as supporting flexible substrate, thus providing additional feature for future integration.

Quantum dots (or any other nanostructures with dimensions of the same order of the emission wavelength in the device) can be used to disperse the light coming from the device itself, if the application requires it.

Using QD-based dielectric materials either of a single material or of a core-shell structures can in principle offer numerous ways to tune the wavelength of the light emitted; however, obtaining a good nanoparticle dispersion within the matrix and the spatial arrangement of the QDs to limit aggregation still remain an important challenge, which prevents a full development and exploitation of this approach. In addition, QDs can be also engineered to improve the mechanical properties of the composites.

### 4.2. Photonic Crystals and Periodic Structures

Photonic crystals are artificially made (hetero)structures where the index of refraction *n* is modulated with a period comparable to the wavelength of light in the material [119,120]. Introducing such periodicity in the structure leads to the formation of a photonic bandgap, thus rendering this structure a photonic insulator in the gap wavelength region, where consequently the light propagation is inhibited, and no absorption loss can significantly change the light propagation even outside the bandgap. A photonic crystal is capable of modifying how the light propagates and interacts with a material, often giving rise to functional and unusual properties such as negative refraction in the proximity of the photonic bandgap [121], strong light confinement in cavities [122] and “*slow*” light as a result of the bandgap formation and crucial in optical buffering [123]. Currently, these structures can be routinely produced with conventional microfabrication techniques, thus holding unlimited potentials to manipulate light.

Among these structures, distributed feedback (DFB) geometries are worthy of particular attention as they offer high reflectivity, long gain lengths, output wavelength purity, stability and high optical confinement, leading to very low operation thresholds [81].

Gwinner et al. integrated a photonic structure made of tantalum peroxide (Ta_2_O_5_) in a top-gate/bottom-contacts OLET based on F8BT, where the emitted light is efficiently coupled with the resonant modes of the DFB waveguide. Moving the recombination zone placed above the waveguide within the channel through appropriate bias modifies the electroluminescence spectrum of the device. Further, by combining this strong two-dimensional confinement in the waveguide structure with a totally reflecting silver gate electrode and an optimized gate dielectric thickness, they were also able to eliminate optical losses at metal electrodes [124].

Namdas et al. proposed a dielectric multilayer structure made of a series of 3 pairs of alternating high- and low-refractive index SiO_2_/SiN_x_ layers of quarter wave thickness deposited on doped *n*-type silicon to enhance the overall device brightness of a SuperYellow-based OLET, where the stack is optimized to act as a reflector at SY emission wavelength. From a dielectric point of view, the capacitance of the multilayer stack is rather low (~10 nF/cm^2^), with very little effect on the electrical performance (no shift in threshold voltage and device driven at high voltages) [125].

**Figure 11:**
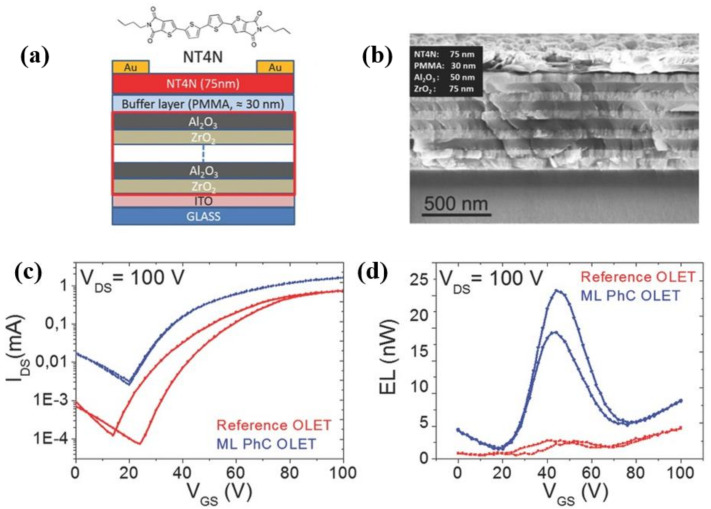
Photonic-crystal as dielectric layer in organic light emitting transistor. (**a**) Simplified schematics of the photonic crystal integrated in the OLED structure, with (**b**) corresponding SEM image of the stack, where alternating ZrO_2_ and Al_2_O_3_ layers are reported. (**c**) *n*-type saturation transfer characteristic and (**d**) electroluminescence (through the substrate) showing a 6-fold increase in the emitted power. Reproduced from [126], © (2020) John Wiley and Sons.

In 2017, Natali et al. integrated a transparent multilayer photonic crystal (ML-PhC), made of alternating layers of high-*k* oxides (ZrO_2_ and Al_2_O_3_) as dielectric in standard top-contacts/bottom-gate ambipolar single-layer organic light emitting configuration [126], as shown in Figure 11a,b. Such photonic structure is capable of modulating the optical characteristics of the emitted light as well as improving the electrical performance of the devices thanks to the high-*k* dielectric properties of the stack (×6 in optical power emitted, ×25 increase in source-drain current in ambipolar limit behavior as shown in Figure 11c,d). Further, it is possible to control the location of the area where emission takes place, and consequently device brightness (×4 in ambipolar conditions).

The fabrication of such structures is fully compatible with in-plane OLET geometry through for example standard lithographic techniques and simpler soft-lithography methods (e.g., micromolding, embossing). Particular attention might be required if these dielectric structures are to be used in top-gate configuration, where any fabrication process and conditions is expected not to interfere with the underlying organic materials already deposited.

Engineered multilayer heterostructures can also be used to tune the wavelength of the emission in organic light emitting transistors, while preserving their dielectric properties.

**Figure 12:**
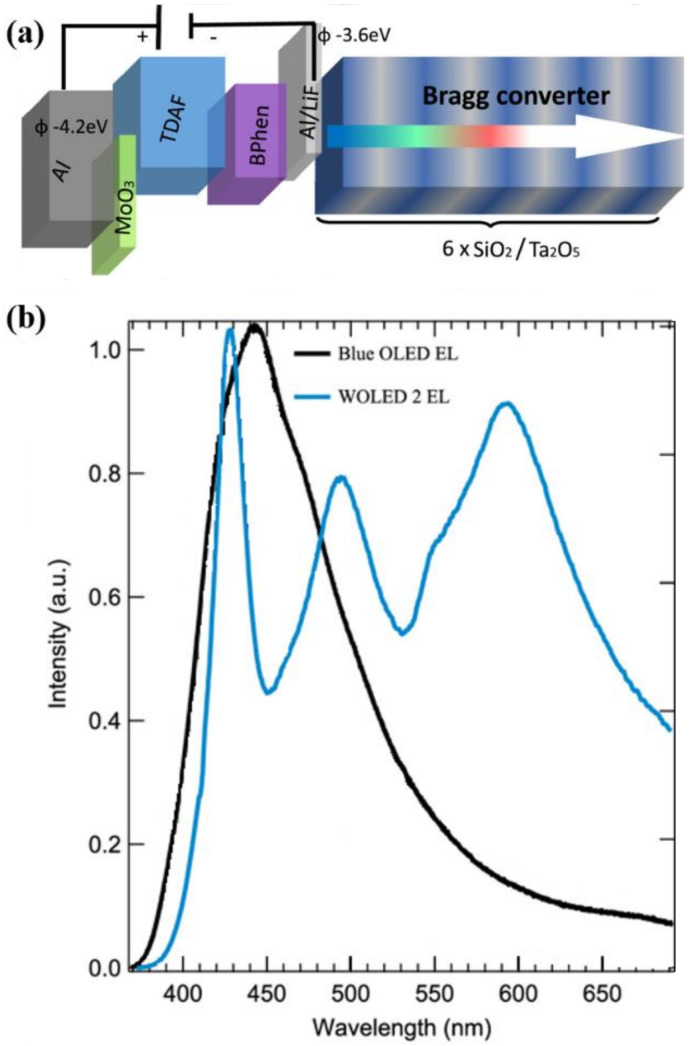
Tuning light emission with DBR in organic light emitting diodes. (**a**) Schematic of the Bragg WOLED concept, where electroluminescence from an emissive layer (TADF) occurs through the Bragg modes of a dielectric DBR. (**b**) Normalized electroluminescence spectra of the OLED (black) before and (blue) after the DBR integration. Reproduced from [127], © (2019) American Chemical Society.

Daskalakis et al. have recently for example shown that a Distributed Bragg Reflector (DBR), consisting of six pairs of two alternating layers with different index of refraction (low-*n*: SiO_2_, high-*n:* Ta_2_O_5_) and coupled with an organic light emitting diode, can act as a high-reflectivity non-absorbing narrow-band mirror (Figure 12a). Constructive interference of the reflected light at the difference interfaces within the multilayer structure enables highly transparent Bragg modes localized within the multilayer structure and its interfaces, with the resulting enhancement and outcoupling of the light at selected wavelengths. This results in white emission starting from a blue OLED (sub-100 nm thick blue single-emissive layer), as shown in Figure 12b. It was also demonstrated that it is possible to tune the color temperature of the white light emission through the DBR geometry and parameters, thus offering great versatility in the optimization of white-light emission spectra which is of relevant interest in for example lighting applications (indoor vs. outdoor illumination) [127]. Although dielectric properties of the DBR are not reported in the work, this structure is expected to have a rather large dielectric permittivity (between 10 and 15), considering the proposed stack includes Ta_2_O_5_ (*k*~23–25), and thus it can be successfully used as a dielectric layer in organic light emitting transistors.

Wu et al. demonstrated that photonic crystals, when coupled with transition metal dichalcogenides (TMD) two-dimensional layers, can be used to enhance not only the light signal (by a factor of approximately 60) but also strongly pattern the emission on the subwavelength spatial scale [128]. In particular, coupling DBR with highly efficient ambipolar organic light emitting devices holds huge potentials for the demonstration and thus development of electrically pumped organic laser [129].

These architectures are compatible with the OLET planar structure and they can be precisely tailored to fulfil optical requirements in the device. Thus, they can serve both as dielectrics as well as optically active material for light manipulation, thus opening the way to broaden the fields of applications for this class of devices, including optically active designing biosensors [130], photonic components [131] and photo-switchable devices [132].

### 4.3. Dielectric Metamaterials and Metasurfaces

Light manipulation can be also realized through metamaterials (3D) and metasurfaces (2D), both artificial nanostructured interface with subwavelength thickness capable of manipulating light by spatially arranged meta-atoms. Generally, those meta-atoms are nanoscale plasmonic or dielectric structures (e.g., nanoantennas), which are capable of strongly and directly modifying some properties of the light (and its associated electromagnetic field), such as polarization, phase and amplitude, through light interaction phenomena such as plasmonic resonances, Mie resonance, Pancharatnam–Berry phase. Depending on materials, metasurfaces can be either be dielectric or plasmonic. Plasmonic metasurfaces comprise metallic meta-atoms and their optical response results from the plasmon resonances between the metallic nanoscale objects and the light. In case of dielectric metasurfaces, the material unit is a dielectric resonators with high refractive index (e.g., Si, Ge, Te) and for which the optical response in based on the interaction of light with electric and magnetic dipole responses based on Mie resonances.

Metamaterials and metasurfaces can be designed, developed and fabricated to efficiently control some of the light characteristics, such as polarization, dispersion, amplitude, wavelength (color) and phase [133,134] with many possible effects including (*i*) loss-free materials overcome the absorption loss of the materials leading to near-unity transmission [135] and (*ii*) modulate refraction and transmission efficiency at specific wavelength [136,137,138]. Technological advancement in nanoscale fabrication methods, has now made metamaterials and metasurfaces technologically viable and experimentally possible, although some processes are still quite complex and expensive.

Sainidou et al. have shown that using planar and periodic arrays of small dielectric particles (alumina immersed in silica) can induce light resonant phenomena, which, in the limit of normal light incidence, leads to total reflection at a resonance wavelength slightly larger than the period for the non-absorbing particles [139]. In particular, it is suggested the possibility to tune the enhancement of the field intensity by tuning the characteristics dimension of the particles (the smaller the particles, the larger the enhancement). Similar properties have also been demonstrated for aligned dielectric cylinders [140].

**Figure 13:**
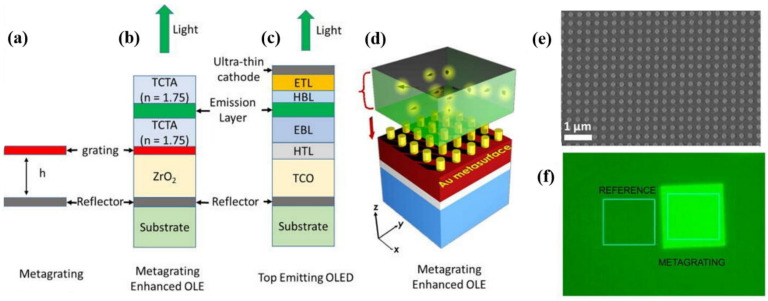
Metagrating enhanced organic light emitting diode structure. (**a**–**c**) Schematic layer structure of a 2D metagrating placed above a metal reflector with its implementation in a light emitting device and its (**d**) 3D rendering schematics. (**e**) SEM image of metagrating consisting of Au cylinders and (**f**) bright emission image (filtered at λ = 520 nm) demonstrating the light enhancement in case of a metagrating integrated device. Reproduced from [141], © (2020) AIP Publishing.

Xu et al. proposed to use a reflecting metagrating, based on a combination of metallic and dielectric materials to enhance the outcoupling light emitted in a light emitting film (which is of potential relevance for both organic light-emitting transistors and diodes) [141]. Figure 13 shows the two-dimensional metagrating placed on top of a metal reflector, separated by a thick layer of high-*k* ZrO_2_, and where the emissive layer (Alq: hydroxyquinolinato aluminum) is sandwiched between two transparent organic transporting layers (TCTA) and where the light is extracted from the top, through an ultra-thin silver cathode. Figure 13d,e show the 3D rendering of such structure and the scanning electron microscope (SEM) image of the metagrating top surface, where the periodic arrangement of Au pillars is visible (diameter: 180 nm, height: 50 nm, pitch: 320 nm). Optical image taken with a green pass-filter in Figure 13f demonstrates the light enhancement obtained in such structures, where the degree of improvement can be tuned through materials choice and device geometry.

While in this case ZrO_2_ is used as a transparent conducting oxide acting as the anode in the device, this can be easily used as dielectric layer in organic light emitting transistors. In fact, ZrO_2_ has for example a dielectric permittivity larger than 30, which would at the same time also ensure the low-bias OLET operating conditions and in some instances can also be deposited by solution-processed technique, thus enabling truly flexible device platform. Further, such structure has all the potential to be monolithically integrated in OLET architecture.

Metasurfaces has also been proposed by Joo et al. to develop a full-color, high-brightness OLED where the metasurface is engineered to behave as a tunable back-reflector. Introducing nanopatterned Ag metasurface mirror specifically for each color (red, green and blue) allows for color tunability of the light emitting device over the entire visible spectrum with improved luminance efficiency and color purity [142]. Such approach, although technologically quite challenging, is expected to be of great impact on the design and fabrication of OLED-based pixels for ultrahigh-density display applications.

## 5. Conclusions and Outlook

Organic light emitting transistors hold great potential as light sources in the field of flexible and wearable applications, and the research community could benefit from extensive research and solutions already developed in large part for the OLED field. Achieving low-bias high-performance OLET devices is the key factor in enabling the full development and exploitation of the overall technology platform, and in this context, implementing high-*k* dielectric materials represents a very promising strategy. Such device platform is of relevant interest to many fields, including light sensing, display technology and lasing, many of which are fully or partially shared with the OLED community.

It is important also to recall here that technology based on organic light emitting diodes has become a commercially viable platform, currently dominating, for example, the display market, from mobile to large scale TVs, after about 40 years of intensive research. Several decades of academic as well as R&D efforts have allowed OLED platform to become be a key enabling technology in many fields; let us just consider the extensive research effort invested by laboratories worldwide and enormous assets from industrial players like Samsung or LG Electronics in the field of displays, just to cite a few.

Organic light emitting transistors are also possible successful candidates to develop electrically pumped organic lasers, since they can achieve high current densities and high quantum efficiency (i.e., multilayer structures), ambipolarity behavior reducing losses from electrons and holes charge density mismatch, well-defined recombination zone and reduced losses due to limited quenching phenomena (such as exciton-charge in proximity of the electrodes). Future development strategies might include split-gate [127] and overlapping split-gate structures [143], where hole and electron currents can be independently controlled to achieve a balanced transport. Coupling with resonating photonic structures capable of amplifying the signal can further increase the possibility for injection lasing to occur. Although still lacking major enabling breakthroughs in this context, OLET holds tremendous potentials for both fundamental studies as well as in terms of application.

As anticipated in the introductory part and through some of the examples shown in the manuscript, a display based on organic light emitting transistors surely represents a very promising, complementary and potentially competitive technology platform compared to OLED, which is already wide available in the consumer market. As said, OLET can bring some specific characteristics including high degree of integration, *pin*-hole and *shorts*-free which can in principle increase manufacturing yield, less stringent requirement at the backplane level along with an overall simplified pixel structure. The year 2016 has seen the showcase at the same time of two distinct Active-Matrix OLET (AM-OLET) display prototypes at Society for Information Display Week in San Francisco (USA). ETC srl (Italy) [144] and NVerPix (USA) [145] have used a conventional planar OLET and a *v*-OLET to demonstrate active matrix addressing in red and green AM-OLET, respectively, on a mobile display size scale. Undoubtedly, this represents an important *proof-of-principle* of the potentials of the OLET platform in the field of active matrix display; nevertheless, a real implementation is yet to come. A simplified OLET structure can potentially reduce fabrication cost, improve yields and simplify the manufacturing process through the adoption of a simplified pixel structure.

Although brightness level and driving voltages might be comparable to currently available OLED alternatives, still several important issues are not addressed and understood. While many materials are of interest for both light emitting platforms and therein applied, the intrinsic difference between OLET and OLED (transistor vs. diode) leaves largely unexplored fundamental parameters and/or mechanisms such as power consumption, lifetime and shelf-life, color stability under stress. Light outcoupling and losses, device stability and encapsulation methods for flexible and wearable displays are also important.

Given the already available knowledge in the diode community, this process can be in principle relatively fast (given the appropriate resources).

OLET planar geometry renders it also compatible with monolithic integration in more complex architectures; whether a grating or a photonic crystal, these structures can be engineered precisely for potentially all visible wavelength to fulfil requirement based on the emission of the device.

## Figures and Tables

**Table 1:** OLET vs. OLED features. Summary of the features of organic light emitting transistors and organic light emitting diodes in terms of charge transport, device architecture and light emission properties.

	Organic Light Emitting Transistor (OLET)	Organic Light Emitting Diode (OLED)
Device architecture	transistor characteristics	diode characteristics
Charge transport	horizontal µm-scale transport (field-effect)	vertical nanoscale transport (tunneling between layers)
Device structure	electrodes: 3 (source, drain, gate) no transparent electrode (light in channel)	electrodes: 2 (anode, cathode) at least one transparent electrode to extract light
	active layer: single or multilayer structure capable of conducting (field-effect) holes and electrons and emitting light	active layer: vertical stack including charge-transport, charge-injection and emissive layers
	dielectric layer: to isolate gate and electrodes and enable field-effect; it prevents shorts	no dielectric layer
Light emission	light occurs in the channel; emission area and brightness can be spatially tuned through bias	light is extracted through one (or two) transparent electrode(s); brightness level can be tuned through diode current

**Table 2:** Summary of the electrical and optical parameters of organic light emitting transistors.

**Electrical**	field-effect mobility(*µ_e_*, *µ_h_*)	mobility of charges (electrons, holes) upon field-effect (can be calculated from Equation (4))
	threshold voltage (*V_th_*_,*e*_, *V_th_*_,*h*_)	voltage corresponding to channel conduction onset (can be calculated from Equation (4))
	ON/OFF ratio	$\frac{I_{D}\left(@V_{D}=V_{G}\;=V_{max}\right)}{I_{D}\left(@V_{D}\;=V_{G}=0\right)}$ high values ensure transistor switching behavior/conductance modulation
**Optical**	Electroluminescence (EL)	device light output upon bias (vs. Photoluminescence, PL: light output upon optical excitation)
	External Quantum Efficiency (EQE)	$\eta_{ext}=\gamma \;\;\eta_{S/T}\;\phi_{PL\;}\;\eta_{out}$ *γ*: number of excitons formed/number of charges in the device *η_S/T_*: spin multiplicity of recombining exciton (singlet/triplet) *φ_PL_*: luminescence quantum yield of the exciton formation layer *η_out_*: light outcoupling efficiency of the device
	Luminance	luminous light intensity projected on a given area and direction
	Brightness	perception of luminance following interaction with human cornea
